# Antioxidant, antibacterial activity, and phytochemical characterization of *Melaleuca cajuputi* extract

**DOI:** 10.1186/s12906-015-0914-y

**Published:** 2015-10-24

**Authors:** Nazeh M. Al-Abd, Zurainee Mohamed Nor, Marzida Mansor, Fadzly Azhar, M. S. Hasan, Mustafa Kassim

**Affiliations:** Department of Parasitology, Faculty of Medicine, University of Malaya, 50603 Kuala Lumpur, Malaysia; Department of Anesthesiology, Faculty of Medicine, University of Malaya, 50603 Kuala Lumpur, Malaysia; Department of Chemistry, Faculty of Science, University of Malaya, 50603 Kuala Lumpur, Malaysia

**Keywords:** *Melaleuca cajuputi*, Antioxidant capacity, Total phenol content, Gas chromatography/mass spectrometry (GC/MS), Liquid chromatography/mass spectrometry (LC/MS)

## Abstract

**Background:**

The threat posed by drug-resistant pathogens has resulted in the increasing momentum in research and development for effective alternative medications. The antioxidant and antibacterial properties of phytochemical extracts makes them attractive alternative complementary medicines. Therefore, this study evaluated the phytochemical constituents of *Melaleuca cajuputi* flower and leaf (GF and GL, respectively) extracts and their antioxidant and antibacterial activities.

**Methods:**

Radical scavenging capacity of the extracts was estimated using 2,2-diphenyl-2-picrylhydrazyl and Fe^2+^-chelating activity. Total antioxidant activity was determined using ferric reducing antioxidant power assay. Well diffusion, minimum inhibitory concentration, and minimum bactericidal concentration assays were used to determine antibacterial activity against eight pathogens, namely *Staphylococcus aureus, Escherichia coli, Bacillus cereus, Staphylococcus epidermidis, Salmonella typhimurium, Klebsiella pneumonia, Streptococcus pneumoniae, and Pasteurella multocida.* We identified and quantified the phytochemical constituents in methanol extracts using liquid chromatography/mass spectrometry (LC/MS) and gas chromatography (GC)/MS.

**Results:**

This study reports the antioxidant and radical scavenging activity of *M. cajuputi* methanolic extracts. The GF extract showed better efficacy than that of the GL extract. The total phenolic contents were higher in the flower extract than they were in the leaf extract (0.55 ± 0.05 and 0.37 ± 0.05 gallic acid equivalent per mg extract dry weight, respectively). As expected, the percentage radical inhibition by GF was higher than that by the GL extract (81 and 75 %, respectively). A similar trend was observed in Fe^2+^-chelating activity and β-carotene bleaching tests. The antibacterial assay of the extracts revealed no inhibition zones with the Gram-negative bacteria tested. However, the extracts demonstrated activity against *B. cereus, S. aureus*, and *S. epidermidis*.

**Conclusions:**

In this study, we found that *M. cajuputi* extracts possess antioxidant and antibacterial activities. The results revealed that both extracts had significant antioxidant and free radical-scavenging activity. Both extracts had antibacterial activity against *S. aureus*, *S. epidermidis*, and *B. cereus*. The antioxidant and antimicrobial activities could be attributed to high flavonoid and phenolic contents identified using GC/MS and LC/MS. Therefore, *M. cajuputi* could be an excellent source for natural antioxidant and antibacterial agents for medical and nutraceutical applications.

## Background

The cells of living organisms generate free-radicals as a result of pathophysiological and biochemical processes in response to factors such as environmental pollutants, radiation, chemicals, and toxins. This creates an imbalance in the formation and neutralization of prooxidants that subsequently seek stability through electron pairing with biological macromolecules such as proteins, lipids, and DNA, leading to oxidative stress in the physiological system [1]. Furthermore, these effects lead to lipid peroxidation as well as protein or DNA damage or both in human cells. Moreover, the cellular damage consequently lead to aging and several chronic diseases such as cancer, diabetes, and atherosclerosis as well as cardiovascular, inflammatory, and other degenerative diseases in humans [1]. The ability of certain phytochemical extracts to inhibit or delay the oxidation of other molecules by suppressing the initiation or propagation of oxidizing chain reactions have made them active alternatives in complementary medicine. These naturally occurring antioxidant chemicals have been reported to be composed of phenolic (such as flavonoids, phenolic acids, and tocopherols) and nitrogen compounds (alkaloids, chlorophyll derivatives, amines, and amino acids) as well as carotenoids and ascorbic acid [2]. In fact, phytochemical extracts containing constituents such as plant-derived vitamins, flavonoids, alkaloids carotenoids, terpenoids, polyphenols, and phenolic compounds such as caffeic, vanillic, ferulic, and ellagic acids have been reported to exhibit antioxidant and anticancer activities [3].

Although chemically synthesized antioxidant compounds such as butylated hydroxytoluene and hydroxyanisole have been used for several decades, the safety of their continued use is currently being questioned due to reports of their carcinogenicity [4]. Therefore, alternative effective antioxidants that have benign or minimal side effects are highly needed.

Infectious diseases caused by microorganism are a major cause of mortality and morbidity in humans. Although several antibiotics have been developed to manage these diseases with optimum efficacy, their mismanagement and maladministration, as well as microbial mutation have led to the emergence of drug-resistant strains. As a result, over the past decades, antibiotics that are known to cure specific diseases have lost their effectiveness. Therefore, the search for new antimicrobial drugs from natural sources is warranted.

Traditional medicine practices in ancient human civilizations worldwide have demonstrated that plants are one of the most promising sources of effective medicinal agents. Therefore, scientific studies have been carried out on the antimicrobial activities of plant extracts against different types of microorganisms, which have resulted in the development of alternative plant-based antimicrobial drugs.

Numerous phytochemical extracts have been evaluated in the process of searching for plant-based antimicrobial agents and some recently reported studies include those on *Syzygium gratum, Justicia gangetica*, and *Limnocharis flava* [5], *Buglossoides purpurocaerulea* [6], Nymphaea nouchali [7], and Polygonum hydropiper [8]. *Melaleuca cajuputi* is commonly known as the Gelam tree and is used to cure cholera as well as muscle and joint pain in folk medicine. It is a member of the Myrtaceae family with reported anti-inflammatory [9], anticancer [10], hepatoprotective [11], and anthelmintic activities [12]. Studies have revealed the antibacterial activity of essential oils of *M. caguputi* against Gram-positive and Gram-negative bacterial strains in the disc diffusion and minimum inhibitory concentration (MIC) assays. The extracted oil inhibited the growth of *Enterococcus faecalis, Escherichia coli*, *Klebsiella pneumoniae*, *Pseudomonas aeruginosa*, *Salmonella enterica*, *Staphylococcus aureus,* and *Streptococcus pyogenes* [13, 14].

In this study, we evaluated the *in vitro* antioxidant and antibacterial activities of methanolic extracts of the leaves and flowers of *M. cajuputi*. In addition, we analyzed the phytochemical constituents of the extracts using liquid chromatography (LC)/mass spectrometry (MS) and gas chromatography (GC)/MS.

## Methods

### Chemical and reagents

Methanol, sodium hydroxide (NaOH), iron (II) sulfate (FeSO_4_), iron (II) chloride (FeCl_2_), sodium nitrite, iron (II) chloride (FeCl_3_), chloroform, hydrochloric acid (HCL), β-carotene, quercetin, chloragenic acid, tripyridyl-s-triazine (TPTZ), butylated hydroxytoluene (BHT), propyl gallate, 2,2-diphenyl −1- picrylhydrazyl (DPPH), linoleic acid, Tween 20, Folin-ciocalteu reagent, acetate buffer, ferrozine, ethylenediaminetetraacetic acid (EDTA), ascorbic acid, and all other reagents and solvents used in this study were of analytical grade purchased from Merck (Merck, Darmstadt, Germany).

### Plant materials

The plant material used in this study was collected from Kedah State, Malaysia, in September 2013, and Identity was confirmed at the Herbarium of Rimba Ilmu, Institute of Biological Sciences, University of Malaya, Kuala Lumpur with voucher number KLU048231.

### Preparation of methanolic extracts

The leaves and flowers of the Gelam tree (*M. cajuputi*) were washed separately with distilled water, ground to a powder, and then dried in the shade for seven days. The methanolic extracts were prepared by adding 100 g of either the Gelam leaves (GL) or Gelam flower (GF) powder to 1 L of absolute methanol in a conical flask and leaving it for 72 h at 25 °C. The mixtures were then filtered using Whatman filter paper (No: 1) to obtain the extract and this procedure was repeated thrice, followed by *in vacuo* concentration at 40 °C using a rotary evaporator to obtain the GF and GL extracts.

### GC/MS analysis

The GC/MS analysis of the methanol extract was carried out by sonicating a 10-mg sample for 15 min in 2.5 mL of dichloromethane at 40 °C in a sealed vial. Then, 1 mL of the treated extract sample was filtered through a 0.20-μm nylon filter into a standard GC 2-mL vial for analysis. The GC/MS analyses were performed at an ionization energy of 70 eV while separation of the hydrocarbons and other volatile compounds were determined using a GCMS-QP2010 series GC system (Shimadzu, Japan) equipped with a DB-5MS Agilent nonpolar column (30 mm × 0.25 mm, 0.25 mm) (Agilent Technologies Inc., Tokyo, Japan). The oven was initially programmed to run at a temperature of 60 °C for 2 min, followed by an increase of 7 °C/min to 150 °C, with a final hold at 310 °C for 15 min. The injector and detector temperatures were kept at 300 (split) and 310 °C, respectively. The analysis was performed with He as the carrier gas at a linear flow rate of 40 cm/s, and the MS detector was operated at 200 °C while the scan range was from 50–1000 m/z at a rate of 0.50 scan/s. To check the purity of each GC peak, the MS was recorded at various parts of each peak. All compounds were putatively identified using a mass spectral database search (National Institute of Standards and Technology/ Environmental Protection Agency/National Institutes of Health, NIST/EPA/NIH) followed by a comparison with the acquired MS data to determine the degree of matching. The compounds that showed mass spectra with match factors ≥90 % were included on the “positive list” of tentatively identified metabolites.

### LC/MS analysis

The LC/MS experiments were conducted to chemically profile the methanolic extracts. The system used to analyze the samples was comprised of an Agilent 1290 Infinity LC system coupled to an Agilent 6520 Accurate-Mass quadrupole time-of-flight mass spectrometer with dual electrospray ionization (ESI) source. The LC separations were performed using a 2.1 mm (i.d) Narrow-BoreSB-C18 (length 150 mm, particle size 3.5 mM) analytical column. The LC parameters used were: autosampler temperature, 25 °C; injection volume, 0.5 μL; column temperature, 25 °C; and flow rate, 0.4 mL/min. A gradient system consisting of solvents A (0.1 % formic acid in water) and B (0.1 % formic acid in acetonitrile) was employed. The mass spectra data were acquired using an ESI capillary voltage of (+) 4000 V and (−) 4000 V in the positive and negative ion modes, respectively with the fragmentor at 125 V. For the other conditions, the liquid nebulizer was set to 45 psi, the nitrogen drying gas was set at a flow rate of 10 L/min with the drying gas and vaporizer temperatures maintained at 300 °C, and the ionization interface was operated in both positive and negative modes. The data were acquired at a rate of 1.03 spectra/s with a stored mass range of 100–3200 and 115–3200 m/z for the positive and negative modes, respectively. The data were collected using the Agilent Mass Hunter Workstation Data acquisition software. LC/MS data files were processed using the Agilent Mass Hunter Qualitative Analysis B.05.00 software. Feature finding was achieved by using the molecular feature extraction and correlation algorithms, which located the groups of covariant ions in each chromatogram. In the positive-ion mode, this included adducts H^+^, Na^+^, K^+^, and NH_4_^+^ and in the negative-ion mode, adducts H^−^ and Cl^−^.

### Total flavonoid assay

The total flavonoid content of the methanolic extracts was determined photometrically using the aluminum chloride (AlCl_3_) assay [15]. Briefly, a 1-mL aliquot of each extract (1 mg/mL) or standard solution of quercetin (31.5, 62.5, 125, 250, 500, and 1 mg/L) was added to a volumetric flask (10-mL) and diluted with 4 mL double distilled water at time 0. Then, 0.3 mL of 5 % (w/v) sodium nitrite (NaNO_2_) was added and after 5 min, 0.6 mL AlCl_3_ (10 %) was added. At 6 min, 2 mL of sodium hydroxide (NaOH, 1 M) was added to the mixture, and the final total volume was made up to 10 mL with double-distilled water. The solution was mixed completely, and the absorbance was measured against a prepared reagent blank in triplicate at 430 nm. The total flavonoid content was expressed as quercetin equivalents in mg/100 g of dry extract weight.

### Total phenolic content (TPC)

The TPC of the methanolic extracts was determined using the Folin-Ciocalteu reagent (Sigma-Aldrich Chemical Co., St. Louis, MO, USA) as previously described by Kim et al. [16], with slight modifications. Briefly, 100 μL of each extract or standard solution of gallic acid (16–1000 μg/mL in 80 % methanol) was mixed with 200 μL of Folin-Ciocalteu reagent, followed by 2 mL of deionized water and 1 mL of 15 % sodium bicarbonate (Na_2_CO_3_). Then, the mixture was incubated for 120 min at room temperature, and the absorbance was measured at 765 nm in triplicate using an ultraviolet (UV)-Visible (Vis) spectrophotometer (GBC, Cintra 40). The total phenolics were quantified using a calibration curve constructed from measurements of the standard gallic acid concentrations and expressed as mg gallic acid equivalent (GAE) per mg of extract weight.

### Determination of antioxidant activity using DPPH radical scavenging

The antioxidant activity of the extracts was measured by determining the hydrogen donating or radical scavenging ability, using the stable radical, DPPH as reported previously [17]. An aliquot (120 μL) of 0.25 mM DPPH solution in methanol and 30 μL of each extract at increasing concentrations (31.3, 62.5, 125, 250, 500, and 1000 μg/mL) were mixed vigorously together and left at room temperature in the dark. The absorbance was measured at 518 nm after 30 min against different concentrations of the extracts in methanol as blanks and DPPH in methanol without extract as the control. The standard synthetic antioxidant, butylhydroxytoluene was used as the positive control. The percentage antiradical activity (AA%) of the extracts was calculated using the following formula [15],

$$ AA\%=\left(100-\left(\frac{Ab{s}_{sample}- Ab{s}_{blank}}{Ab{s}_{control}}\right),\times, 100\right) $$

Where, Abs_sample_, Abs_blank_, and Abs_control_ are the absorbance values of the extract, blank, and control samples, respectively.

### β-Carotene bleaching test

The β-carotene bleaching test was used to evaluate the antioxidant activities based on the β-carotene lenolate system model [18], with slight modifications. Briefly, 1 mL of β-carotene solution (0.2 mg/mL in chloroform) was added to 0.02 mL of linoleic acid and 0.2 mL of 100 % Tween 20. Then, 5 mL samples of this emulsion were transferred into test tubes containing 0.2 mL of test samples in 80 % methanol at increasing concentrations (62.5, 125, 250, 500, and 1000 μg/mL). These mixtures were then incubated in a water bath at 40 °C for 120 min. All determinations were performed in duplicate and the mean values calculated. The absorbance was measured at 470 nm using a Perkin–Elmer Lambda 40 UV/Vis spectrophotometer against a blank consisting of the emulsion without β-carotene. The measurements were carried out at initial and final times (t = 0 and 120 min, respectively) with propyl gallate as the positive control. The AA% was measured and expressed as the percentage of inhibition of β-carotene oxidation using the following equation:

$$ AA\% = \left[\left(\frac{A{S}_0-A{S}_{120}}{A{C}_0-A{C}_{120}}\right)\right]\times 100 $$

Where AS_0_ and AS_120_ are absorbance values of the samples and AC_0_, and AC_120_ are the controls at 0 and 120 min, respectively.

### Fe^2+^-chelating activity assay

The Fe^2+^-chelating activity of GL and GF extracts was measured as follows: The extract was treated with ferrozine (5 mM), which reacted with the divalent iron to form a stable and highly water-soluble magenta complex species. After 10 min at room temperature, the absorbance of the Fe^2+−^ferrozine complex was measured at 562 nm. The Fe^2+^-chelating activity of the extract was calculated using the following equation:

$$ \%\mathrm{Chelating}\ \mathrm{rate}=\frac{\mathrm{A}0-\mathrm{A}1}{\mathrm{A}0} \times 100 $$

Where, A_0_ and A_1_ are the absorbance values of the control (blank without extract) and in the presence of the extract, respectively.

### Ferric reducing antioxidant power (FRAP) assay

The ferric reducing power of the extracts was assayed based on the blue coloration that developed due to the reduction of ferric iron to the ferrous form as described previously [18]. Extract solutions were prepared by dissolving about 0.1 μg/mL of extracts in ethanol. An aliquot (0.2 mL) of each extract solution was added to a test tube containing 1.8 mL of freshly prepared FRAP reagent that consisted of 2.5 mL of 10 mM TPTZ solution in 40 mM of HCl and 2.5 mL of 20 mM FeCl_3_.6H_2_O in 25 mL of 0.3 M acetate buffer (pH 3.6). The mixture was incubated at 37 °C for 5 min. The spectrometric absorbance was recorded at 593 nm. The reducing power was ascertained by comparing the spectrophotometric absorbance of each sample against a standard curve obtained from Fe_2_SO_4_.

## Antimicrobial activity

### Test organisms

The *in vitro* antibacterial activities of the *M. cajuputi* extracts were evaluated against eight strains that were supplied by the Microbiology Laboratory of the University of Malaya Medical Centre. They comprised of four Gram-positive (*Staphylococcus epidermidis*, MTCC 3615; *Staphylococcus aureus,* RF 122; *Bacillus cereus*, ATCC 11778*;* and *Streptococcus pneumoniae*, ATCC 10015) and four Gram-negative (*Escherichia coli*, UT181; *Salmonella typhimurium*, ATCC 14028; *Klebsiella pneumonia*, ATCC13883; and *Pasteurella multocida*, a clinically isolated strain) bacterial strains. All the strains were stored in the appropriate medium before use.

### Inocula preparation

The colony suspension method was used to prepare the inocula of the test organisms. The bacterial strains were grown on nutrient agar (NA) at 37 °C for 18 h, and then adjusted to a turbidity of 0.5 McFarland standards (10^6^ colony forming units, CFU/mL) based on the optical density (OD) measurement at 620 nm. After being cultured for 24 h on NA, the colonies were collected, and cultured in nutrient broth medium for 24 h at 37 °C. The susceptibility tests were subsequently performed using the NA-well diffusion method.

### Bacterial cultures and disc diffusion assay

The disc diffusion method is a widely acclaimed method used in screening crude extracts for antibacterial activities. In this study, the antibacterial activity was determined based on the method previously described [19] with modifications. Briefly, the crude extract at a concentration of 0.1 g/mL was dissolved in 100 % dimethyl sulfoxide (DMSO, Merck, Germany) and sterilized by filtration using a 0.20-mm Millipore disposable filter (Minisart, Sartorius Biotech, Germany). Autoclave-sterilized (121 °C for 20 min) Mueller Hinton Agar (MHA) medium (BioLab) was used in the disc diffusion assay. A 50-μL sample of the filtration-sterilized plant extract was loaded onto a sterile paper disc (6 mm in diameter), which was then placed on the surface of the agar plate (NA) previously inoculated with the bacteria. A disc prepared under the same conditions with only 50 μL of DMSO was used as a negative control. In addition, a similar disc was loaded with the reference antibiotic (streptomycin) at a concentration of 20 mg of drug per disc and used as described above. Both samples were allowed to diffuse into the agar plates for 1 h and were then inverted and incubated at 37 °C for 18 h. Antibacterial activity was determined by measuring the diameter of the growth inhibition zones (IZs, mm) surrounding each disc. Each assay was performed in triplicate with two repetitions and the results were expressed as average values.

### MIC and minimal bactericidal concentration (MBC) assays

The MIC values, which represent the lowest plant extract concentration that completely inhibits the growth of microorganisms, were determined using a micro-well dilution method as described previously [20]. In addition, the MBC values refer to the lowest concentration of an antibacterial agent required to prevent the growth of a particular bacterium after subculture in an antibiotic-free medium. Briefly, the extracts were dissolved in DMSO at 100 mg/mL, and then twofold serial dilutions were prepared in a 96-well dilution microplate. The antibiotic streptomycin was included as reference agent in each assay while the extract-free solution was used as a blank control. Each well of the microplates contained 40 μL of growth medium, 10 μL of inoculum (10^6^ CFU/ml), and 50 μL of diluted sample extracts. Then, the microplates were incubated overnight at 37 °C. As an indicator of microorganism growth, 40 μL of p-iodo nitro tetrazolium violet (INT) dissolved in water was added to the wells, and the plates were incubated at 37 °C for 30 min. The colorless tetrazolium salt acts as an electron acceptor and is reduced to a red-colored formazan product by biologically active organisms [20]. A Tecan microplate reader (Infinite M200PRO) was used to quantify the OD of the reactants in each well. Where microbial growth was inhibited, the solution in the well remained clear after incubation with INT. The determination of MIC values was performed in triplicate. The MBC of the extracts was determined by subculturing samples from the MIC assay tubes onto NA plates from wells that showed growth inhibition, and then subsequently determining the dilution at which growth was arrested, which was considered the MBC.

### Statistical analysis

The data were expressed as mean ± standard deviation (SD) of triplicate determinations. The half-maximal inhibitory concentrations (IC_50_) values were estimated from the AA% versus concentration plots using a non-linear regression algorithm.

## Results and discussion

In this study, we evaluated the chemical constituents of GL and GF using preliminary GC/MS and LC/MS analysis. Furthermore, we determined their antioxidant and antibacterial activities using various *in vitro* methods.

### GC/MS and LC/MS analysis of GF and GL from *M. cajuputi*

The phytochemical analyses of plant extracts are normally performed using diverse quantitative and qualitative analytical techniques spanning from chromatography to spectroscopy [21]. Previously, K-L Li and S-J Sheu [22] used a micellar electrokinetic capillary chromatographic method to analyze the phytochemical constituents of scute-coptis, a dual herbal combination. Using this method, the researchers identified six scute flavonoids namely baicalin, wogonin 7-*O*-glucuronide, oroxylin A 7-*O*-glucuronide, baicalein, wogonin, and oroxylin A as well as four coptis alkaloids comprising of berberine, palmatine, coptisine, and epiberberine.

In another study, GC/MS was used to characterize the chemical content of *Melaleuca* essential oils [23–25]. Similarly, silica gel chromatography was used to isolate a new chromone from *M. cajuputi* leaf extracts [26]. Using a combination of spectroscopic techniques, the study characterized the newly isolated chromonone as melachromone [26].

Similarly, in this study, preliminary compound identification and quantitation was performed using GC/MS and LC/MS. As expected, flavonoids and alkaloids were consistently present in all samples. Furthermore, the occurrence of terpenoids, saponins, glycosides, and steroids depended on the type of plant part extract analyzed. The occurrence of the identified compounds mentioned in Tables 1, 2 and 3 in this study has been previously reported in *Melaleuca* extracts [27, 28].

**Table 1:** List of major compounds identified from *M. cajuputi* Laef extract

ID	Posibble Compound Name	Class of compound	Mol. formula	Mol mass	Rt time (min)	%
1	3-Cyclohexen-1-ol	Terpenoids	C18H18O	154	6.152	1.08
2	Cyclohexane, 1-ethenyl-1-methyl-2,4bis(1-methylethenyl)	Terpenoids	C15H24O4	204	8.109	1.83
3	Caryophyllene Bicyclo[7.2.0]undec-4ene	Terpenoids	C15H24	204	8.589	3.64
4	Caryophyllene 1,4,8-Cycloundecatriene	Terpenoids	C15H24	204	9.061	2.32
5	Naphthalene	Terpenoids	C15H24	204	9.537	2.13
6	Naphthalene	Terpenoids	C15H24	204	9.619	1.66
7	1H-Cycloprop[e]azulen-7-ol	Terpenoids	C15H24O	220	10.914	2.27
8	Caryophyllene oxide 5-Oxatricyclo[8.2.0.0(4,6)-]dodecane	Terpenoids	C15H24O	220	11.046	2.0
9	Alpha.-Tetralone	Aromatic	C12H13FO3	224	11.308	7.0
10	2-Naphthalenemethano	Sesquiterpene	C15H24O	222	12.325	2.38
11	Spathulenol 1H-Cycloprop[e]azulen-7ol	Sesquiterpene	C15H24O	222	13.636	1.31
12	Ethanone	Phenolic	C10H10O5	210	15.181	2.83
13	3,7,11,15-Tetramethyl-2-hexadecen-1ol $$ (2E)-3	Fatty acid	C20H40O	296	15.708	2.50
14	3-Eicosyne 3-Icosyne	Straight chain	C20H38	278	16.634	0.87
15	4H-1-Benzopyran-4-one	Flavone	C11H10O4	206	16.791	1.38
16	1,4-Naphthalenedione	Aromatic	C11H8O5	220	18.750	4.53
17	4H-1-Benzopyran-4-one	Flavonoids	C11H8O5	220	19.298	6.09
18	Ethanone	phenolic	C16H14O4	234	19.713	8.81
19	Methyl lathodoratin	Flavonoids	C12H12O4	220	20.921	0.57
20	Phytol 2-Hexadecen-1-	Fatty acids	C20H40O	296	21.666	0.57
21	Octadecanoic acid	Fatty acid	C22H44O2	340	27.517	1.31
22	1-Heptacosanol	Straight chain	C27H56O	396	33.437	0.27
23	Squalene	Straight chain alkene	C30H50	410	35.458	2.05
24	1-Heptacosanol	Straight chain	C27H56O	396	33.437	0.27
25	2H,6H-Pyrano[3,2-b]xanthen-6-one	Flavonoids	C18H14O6	326	39.607	0.41
26	Alpha Tocopherol (vit E)	Phenolic	C29H50O2	430	43.406	2.37
27	Sitosterol, Stigmast-5-en-3-ol	Terpenoids	C29H50O	414	43.406	2.37
28	Urs-12-en-28-al	Terpenoids	C30H48O	424	44.485	0.95
29	Dammarane-3,12,25-triol	Terpenoids		562	47.086	0.55
30	Betulin Lup-20(29)-ene-3,28-diol	Terpenoids	C30H50O2	482	47.947	1.15
31	Urs-12-en-28-al	Terpenoids	C30H48O	424	50.881	0.96

**Table 2:** List of major compounds identified from Gelam flower extract

ID	Posibble Compound Name	Class of compound	Mol. formula	Mol mass	Rt time (min)	%
1	3-Cyclohexen-1-ol	Terpenoid	C18H18O	154	6.151	1.07
2	Cyclohexane, 1-ethenyl-1-methyl-2-(1methylethenyl)-4-(1-methylethylidene)-	Terpenoid	C15H24	204	7.482	0.68
3	Copaene	Terpenoid	C15H24	204	7.997	1.66
4	Cyclohexane, 1-ethenyl-1-methyl-2,4bis(1-methylethenyl)	Terpenoid	C15H24O4	204	8.107	2.60
5	Caryophyllene Bicyclo[7.2.0]undec-4ene	Terpenoid	C15H24	204	8.588	6.14
6	1,6-Cyclodecadiene, 1-methyl-5methylene-8-(1-methylethyl)-,	Terpenoid	C15H24	204	8.690	1.49
7	Caryophyllene 1,4,8-Cycloundecatriene	Terpenoid	C15H24	204	9.059	3.16
8	Naphthalene	Aromatics	C15H24	204	9.536	3.26
9	Naphthalene	Aromatics	C15H24	204	9.616	2.66
10	Naphthalene	Aromatics	C15H24	204	9.851	1.97
11	1H-Cycloprop[e]azulen-7-ol	Terpenoid	C15H24O	220	10.906	1.29
12	Caryophyllene oxide 5-Oxatricyclo[8.2.0.0(4,6)-]dodecane	Terpenoid	C15H24O	220	11.176	1.73
13	Alpha.-Tetralone	Aromatics	C12H13FO3	224	11.279	6.24
14	2-Naphthalenemethanol	Aromatics	C15H26O	222	12.318	2.84
15	2-Naphthalenemethanol	Aromatics	C15H26O	222	15.545	0.98
16	Hexadecanoic acid	Fatty acid	C17H34O2	270	17.648	0.60
17	1,4-Naphthalenedione	Phenolic	C11H8O5	220	18.672	1.07
18	4H-1-Benzopyran-4-one	Flavonoids	C16H20O4	276	19.168	2.12
19	Ethanone	Phenolic	C16H14O4	234	19.563	4.34
20	9,12-Octadecadienoic acid (Z,Z)-,	Fatty acids	C19H34O2	294	21.327	1.90
21	Octadecanoic acid	Fatty acid	C19H38O2	298	22.779	0.68
22	1-Heptacosanol	Straight chain	C27H56O	396	40.363	0.85
23	Alpha Tocopherol (vit E)	Phenolic	C29H50O2	430	40.600	0.47
24	Sitosterol, Stigmast-5-en-3-ol	Terpenoids	C29H50O	414	42.438	0.57
25	Sitosterol, Stigmast-5-en-3-ol	Terpenoids	C29H50O	414	43.399	3.88
26	Urs-12-en-28-al	Terpenoids	C30H48O	424	44.480	1.47
27	Urs-12-en-28-al	Terpenoids	C30H48O	424	45.816	4.93
28	Urs-12-en-28-al, 3-(acetyloxy)-,	Terpenoids	C32H50O3	482	47.628	1.49
29	Betulin $$ Lup-20(29)-ene-3,28-diol,	Terpenoids	C30H50O2	442	47.949	3.68
30	Urs-12-en-28-al	Terpenoids	C30H48O	424	48.690	3.00
31	3.beta.-Myristoylolean-12-en-28-ol	Terpenoids	C44H76O3	652	50.431	1.67
32	Urs-12-en-28-al	Terpenoids	C30H48O	424	50.867	2.41

**Table 3:** Chemical composition comparison of methanol extract from flower, and leaves extract of *M. cajuputi* based on LCMS

Phytochemical extract	GF extract		GL extract		RT	m/z
	presence	% Abundance	presence	% Abundance		
Metyrosine	+	0.6	+	0.39	13.072	194.08203
Methylorsellinic Acid, Ethyl Ester	+	0.74	+	8.02	11.18	209.0822
Hydroxyibuprofen	+	1.2	+	0.74	10.759	221.1184
Trans-2, 3, 4-Trimethoxycinnamate	+	0.7	+	11.61	10.386	237.07724
Gingerol	+	0.45	+	0.28	11.768	293.17629
Catharanthine	+	2.1	+	1.0	20.202	371.15374
calicoferol D	+	1.1	+	0.65	21.489	409.31181
Caffeic acid Phenethyl ester (CAPE)	+	18.69	-	-	18.451	283.30206
Aspidin	+	0.65	+	0.59	12.819	459.20282
Cucurbitacin F	+	1.2	+	1.2	20.886	517.31874
Kurilensoside G	+	2.2	+	3.1	20.89	633.33896
1α,22,25-trihydroxy-26,27-dimethyl-23,23,24,24-tetradehydro-24a,24b,24c-trihomovitami	+	9.68	+	9.48	20.869	497.36525

*Rt* Retention time (as min), m/z mass

As it can be seen in Table 1, the GC/MS analysis of the GF revealed that the major compounds are essential oils, characterized by the presence of fatty acids including octadecanoic (0.68 %), hexadecanoic (0.60 %), and 9,12-octadecadienoic acids (1.90 %). Additionally, there were phenolic compounds such as alpha-tocopherol (vitamin E, 0.47 %), ethanone (4.34 %), 1,4-naphthalenedione (1.07 %), and terpenoid compounds such as Urs-12-en-28-al (6.40 %). Furthermore, aromatic compounds such as naphthalene (7.92 %) and alpha-tetralone (6.24 %) were detected. Previous studies have demonstrated that some of the identified compounds in the GF extract such as alpha-tocopherol [29] and hexadecanoic acid [30] possess antioxidant activities.

GC/MS analysis of the GL extract revealed the presence of 31 phytochemical compounds. As shown in Table 2, the methanol extract predominantly contained aromatic compounds such as alpha-tetralone (7 %) and 1,4-naphthalenedione (4.53 %); phenolic compounds such as ethanone (11.6 %); terpenoids such as caryophyllene bicyclo [7.2.0] undec-4ene (3.64 %), naphthalene (3.79 %), and sitosterol (2.37 %); and flavonoids such as 4H-1-benzopyran-4-one (6.09 %). In addition, squalene (2.05 %) and octadecanoic acid (1.31 %) were also present. Furthermore, numerous previous studies have reported the antioxidant activities of some of these compounds [31, 32].

**Fig. 1:**
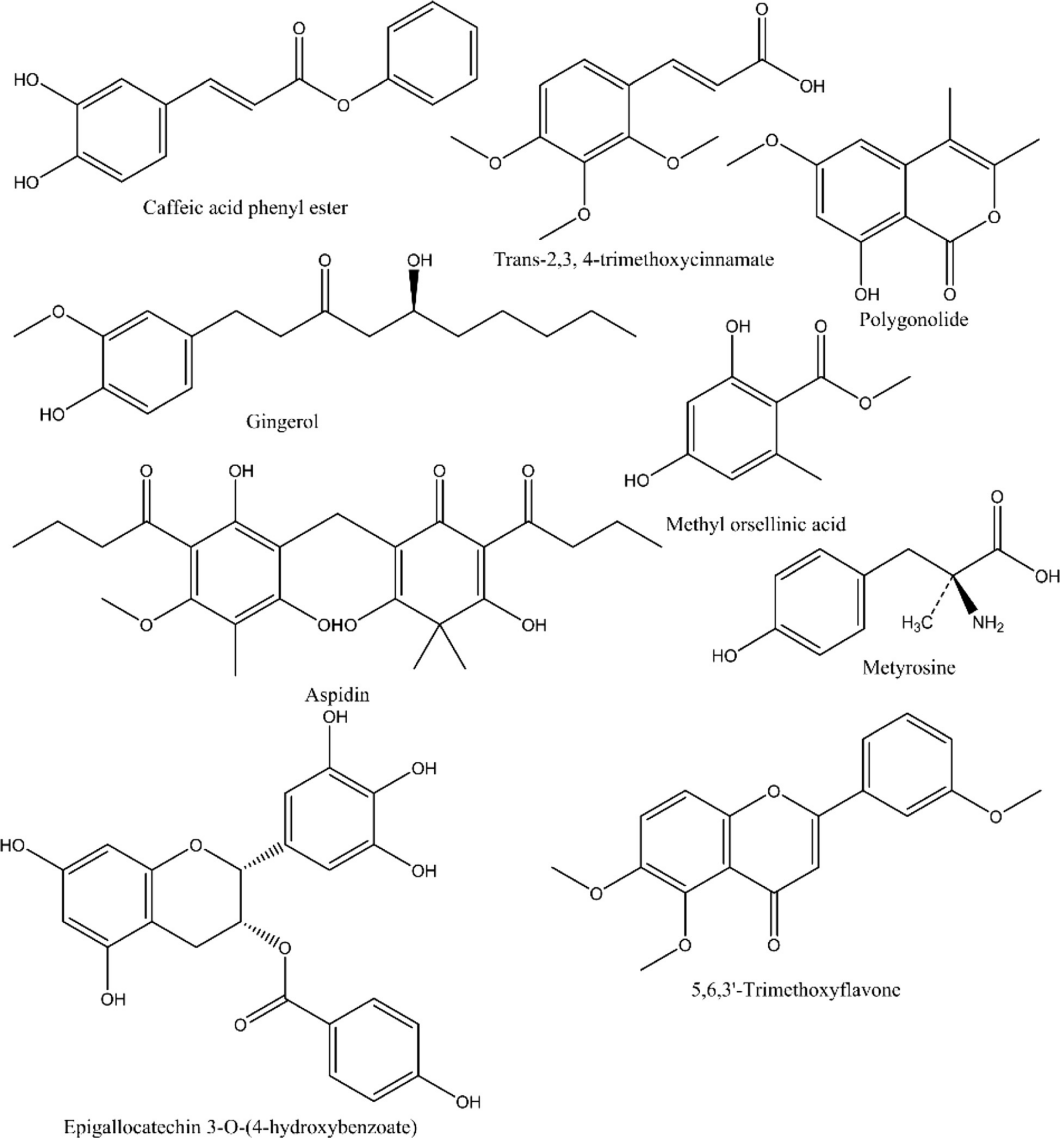
Chemical structure of the observed phytochemical compounds in *M. cajuputi* flower and leaves extracts

The results of the LC/MS analyses are presented in Table 3. Most of the observed compounds were typical hydroxycinnamic acid and phenolic acid derivatives (Fig. 1). The LC/MS analysis of the GF extract indicated the presence of caffeic acid phenyl ester, gingerol, aspidin, methyl orsellinic acid ester, ethyl ester, trans-2,3, 4-trimethoxycinnamate, and metyrosine. In addition, the GL extract contained epigallocatechin 3-O-(4-hydroxybenzoate), 5,6,3'-trimethoxyflavone, metyrosine, gingerol, polygonolide, and trans-2, 3, 4-trimethoxycinnamate.

### Total phenolic, total flavonoids contents, and antioxidant activity

Numerous phenolic metabolites containing an aromatic arene (phenyl) ring with one or more acidic hydroxyl residues attached to it are known to be produced by plants. A previous study showed that compounds such as flavonoid and tannins were among the major phenolic constituents present in plant extracts [33]. The phenolic radicals were reported to be less reactive and with a lower electron reducing potential than the oxygen radicals had [33, 34]. Because of these properties, phenolic compounds are considered excellent radical scavengers. Furthermore, phenolic compounds are able to scavenge reactive oxygen intermediates without invoking further oxidative reactions. Therefore, one of the current standards of phytochemical research is the evaluation of the TPC as a measure of determining the antioxidant activity of extracts. Therefore, the current study evaluated the TPC of the extracts and found that the GF extract showed a higher value than the GL extract did (55 ± 0.05 and 37 ± 0.05 GAE/mg extract dry weight, respectively). A similar trend was also observed with the flavonoids content of both extracts (Table 4, GF > GL extracts). However, it is not surprising that the higher TPC value of the GF confers a stronger antioxidant ability than that of the GL. Therefore, the higher the TPC content of an extract, the higher its antioxidant activity will be. This is because the substituted 5,7,3′,4′-hydroxy flavonoids are believed to possess a very efficient radical scavenging power [35]. This observation was in agreement with the report of a direct relationship between the flavonoid and phenolic contents and the biological activities of plant extracts [36–38].

**Table 4:** A comparison of total phenolic and flavonoids contents

Methanolic Extract	Total phenolic content (GAE/mg dw)	Total flavonoid content (QE/mg dw)
*M. cajuputi* flower extract	55 ± 0.03	19.6 ± 0.4
*M. cajuputi* leaves extract	37 ± 0.02	10.2 ± 0.2

*GAE* Garlic Acid Equivalent

*QE* Qurcetin Equivalent

### DPPH radical scavenging activity

**Fig. 2:**
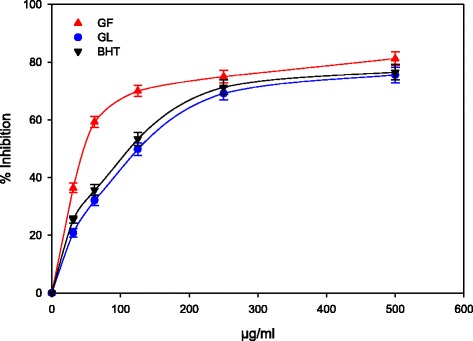
Antioxidant activity of *M. cajuputi* flower and leaves extracts following DPPH radical scavenging assay

The direct and rapid reaction between DPPH radicals and antioxidants has been utilized as a measure of antioxidant activity [39] and a high percentage DPPH radical scavenging of a compound indicates excellent activity. In this present study, the free-radical scavenging activity of the GL and GF methanolic extracts, which was evaluated using DPPH, was found to agree with TPC observation. Therefore, the extract with the higher TPC value also showed a higher percentage DPPH radical scavenging activity (Fig. 2). Furthermore, in agreement with the TPC and flavonoid observations, the percentage radical inhibition by GF extract was also higher than that of the GL extract was (81 and 75 %, respectively). This observation confirmed that both the GF and GL extracts exhibited DPPH radical-scavenging activity concentration-dependently, although only the GL extract showed a scavenging power that was greater than the values obtained with the BHT positive control. For both samples, however, increasing the concentration beyond 250 μg/mL resulted in a negligible increase in the radical scavenging activity (Fig. 2). Based on the calculated IC_50_ values, only the GL extract revealed a higher scavenging effect than the GF extract by demonstrating a lower IC_50_ value (10 μg/mL) than the positive control BHT (13 μg/mL); the GF extract showed an IC_50_ value of 25 μg/mL. Several reports have indicated that free radical scavenging activity is greatly influenced by the phenolic contents of the sample, flavonoid, and the presence of hydroxycinnamic acids such as caffeic acid phenyl ester [40–42]. Similarly, high antioxidant activity was previously reported in *Melaleuca* [43].

### Fe^2+^-chelating activity

**Fig. 3:**
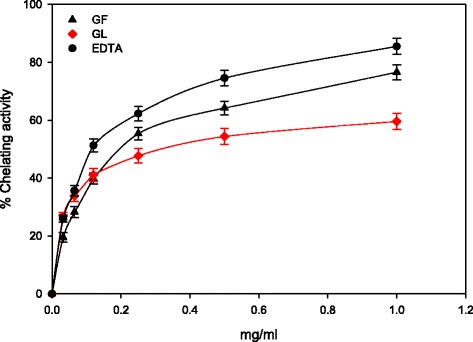
Metal chelating activity of *M. cajuputi* flower and leaf extracts

In cellular lipid peroxidation determination, the Fenton reaction is used in metal chelating activity assays to reduce the concentration of the catalyzing transition metal. This kind of chelating reaction is considered significant in reducing the oxidative stress generated by reactive oxygen species. The chelating effect of the methanolic extracts was around 50 % at an extract concentration of around 0.4 mg/mL, and the chelating activity was concentration-dependent (Fig. 3). As expected, the activity of the GF extract was higher than that of the GL extract was, with maximum chelating activities of ~75 and 59 %, respectively (Fig. 3). The presence of a significant amount of caffeic acid phenyl ester in the GF may have contributed to this activity as these substances have been reported to exhibit strong chelating activity [44].

All the samples tested exhibited a logarithmic increase in chelating power with increasing concentrations up to 0.25 mg/mL and, thereafter, the percentage chelating activity appeared to increase gradually.

### β-Carotene bleaching test

**Fig. 4:**
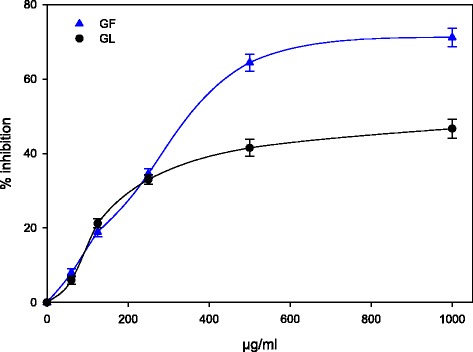
Antioxidant activity of *M. cajuput* flower and leaf extracts determined by β-carotene bleaching test

Carotenoids are among the most common natural pigments and are responsible for most of the red, orange, and yellow coloration of plant leaves, fruits, and flowers [45]. Currently, more than 600 different carotenoid compounds were reported to have been characterized [45]. In animals, carotenoids act as antioxidants. Furthermore, carotenoids have attracted much attention because numerous studies have revealed that their consumption is correlated with a diminished risk for several degenerative disorders including various types of cancer and cardiovascular or ophthalmological diseases [45]. This effect is attributed to their antioxidant activity, which protects cells and tissues from oxidative damage [45]. In this study, all the analyzed samples inhibited the discoloration of β-carotene in a concentration-dependent manner (Fig. 4). Generally, an increase in the percentage inhibition was observed with all the samples tested. As expected, the GF extract was more effective than the GL extract was with inhibition rates of 71 and 47 %, respectively.

### FRAP assay

The FRAP assay is primarily based on the principle of reduction of ferric ions to their ferrous form at a lower pH, which results in the formation of a chromatic ferrous-tripyridyltriazine complex [46]. This assay is considered to be an accurate method for testing the antioxidant power of therapeutic compounds [47]. In this research study, the FRAP antioxidant ability of the *M. cajuputi* extracts in reducing the Fe^3+^–TPTZ reagent was evaluated and both extracts (GL and GF) demonstrated some reducing power with FRAP values of 0.12 and 0.14 μM Fe(II)/g, respectively. The presence of a significant amount of caffeic acid phenyl ester in the GF extract likely contributed to its higher FRAP value than that of GL extract since this compound is known to possess strong FRAP antioxidant activity [44]. Zunjar and colleagues [36] reported that the reducing capacity of a compound may serve as a remarkable indicator of its antioxidant activity and ability to ameliorate oxidative stress by reacting with certain precursors.

### Antibacterial activity

The antibacterial activity of the *M. cajuputi* extracts was evaluated against four Gram-positive (*B. cereus*, *S. epidermidis*, *S. aureus*, and *S. pneumoniae*) and four Gram-negative (*E. coli*, *P. multocida*, *K. pneumoniae,* and *S. typhimurium*) bacteria. The antibacterial activity of the extracts was assessed by determining their IZ, MIC, and MBC values (Tables 5 and 6).

**Table 5:** Antibacterial activity of crude extracts

Inhibition diameter (mm ± SD)								
Sample	*Staphylococcus epidermidis* (Gram + ve)	*Staphylococcus aureus* (Gram + ve)	*Bacillus cereus* (Gram + ve)	*Pasteurllamultocida* (Gram + ve)	*Klebsiella pneumonia* (Gram + ve)	*Streptotococcus pneumonia* (Gram + ve)	*Esherichia coli* (Gram -ve)	*Salmonella typhimurium* (Gram -ve)
*M.cajuputi* Leaves	13.66 ± 0.43	12.33 ± 0.57	6.33 ± 0.33	-	-	-	-	-
*M.cajuputi flower*	17.33 ± 0.36	12.33 ± 0.31	12.33 ± 0.48	-	-	-	-	-
Streptomycin sulfate^a^	20.33 ± 0.38	18.0 ± 0.2	21.0 ± 0.25	21.0 ± 0.05	20.0 ± 0.1	22.0 ± 0.08	15.0 ± 0.1	10.0 ± 0.2

^a^Doses of Streptomycin was 1 mg/ml

-No Activity observed

**Table 6:** MIC and MBC of *M. cajuputi* extracts

	MIC (mg/mL)			MBC (mg/mL)		
SAMPLE	*Staphylococcus aureus*	*Staphylococcus epidermidis*	*Bacillus cereus*	*Staphylococcus aureus*	*Staphylococcus epidermidis*	*Bacillus cereus*
G.L.	12.5	12.5	NA	25	25	NA^**^
G.F.	12.5	25	12.5	50	25	50
Streptomycin*	1.95	1.95	>1.0	1.95	1.95	>1.0

*Doses of Streptomycin was 1 mg/ml

^**^NA No Activity observed

The results revealed that both the GL and GF extracts potently inhibited *S. epidermidis*, *S. aureus*, and *B. cereus* (Table 5). The GF extract was more effective than GL against *S. epidermidis* and *B. cereus,* while both extracts showed comparable activity against *S. aureus*. However, both extracts had no effect against the tested Gram-negative organisms and *P. multocida.* The most susceptible bacteria to the GF extract were *S. aureus*, *S. epidermidis*, and *B. cereus* with MIC values of 12.5, 12.5, and 25 g/mL, respectively. Furthermore, the results showed there were no observed MIC and MBC values against *B. cereus* exposed to the GL extract (Table 6). However, the exposure of *S. aureus* to both extracts resulted in identical MIC, and MBC values 12.5, and 25 mg/mL, respectively.

The MBC for the GF extract against S. epidermidis was 25 mg/mL, which was more effective than the GL extract was at 50 mg/ml. These findings are of great significance, especially in the case of *S. aureus* and *B. cereus* that are well-known for being resistant to numerous antibiotics. In addition, these organisms are capable of producing several types of enterotoxins that can cause septicaemia and several forms of enteritis. In general, the GF extract was found to be active against some species of *Staphylococci* and *Bacilli* while the GL extract was inactive against the tested *Bacilli*. Therefore, the antibacterial activity of the extracts could be correlated with their phenolic and flavonoids contents. KA Hammer, C Carson and T Riley [13] reported a similar observation for the antimicrobial activity of *M. cajuputi* extract against *S. aureus*. In contrast, it was reported that the hexane, dichloromethane, and acetone extracts of *M. cajuputi* leaves showed no activity against *S. aureus*, methicillin-resistant *S. aureus*, *E. coli*, and *P. aeruginosa* [26].

## Conclusions

We attempted to explore the diverse phytochemical efficacy of *M. cajuputi* against several diseases and oxidative stress, by evaluating the antioxidant and antibacterial activities of its GF and GL methanolic extracts. Furthermore, to the best of our knowledge, this is the first comprehensive study of the antioxidant and antibacterial potential of the GF and GL extracts. Generally, the GF extract showed a higher efficacy than the GL extract did. In addition, the TPCs were higher in the GF extract than they were in the GL extract, and these results were in agreement with the percentage radical inhibition results, which were higher for the GF extract than they were for the GL extract. The same trend was also observed in the Fe^2+^-chelating activity, flavonoid contents, and β-carotene bleaching test. Both extracts showed promising evidence of antibacterial activity against *S. aureus*, *S. epidermidis*, and *B. cereus*. Finally, the observed antioxidant and antibacterial activities of these extracts could be attributed to the high content of phenolics and flavonoids identified using LC/MS and GC/MS.
